# Establishing content validity for the migraine Global Impression Item (mGI-I) assessment: a modified single-item migraine symptom severity questionnaire

**DOI:** 10.1186/s12883-022-02626-0

**Published:** 2022-03-18

**Authors:** David Chandler, Marco Navetta, Shweta Shah, Jennifer Cline, Michael Phinney

**Affiliations:** 1 Amgen, 1 Amgen Center Dr, Thousand Oaks, CA 91320 USA; 2 IQVIA, 4820 Emperor Blvd, Durham, NC 27703 USA

**Keywords:** Migraine global impression item (mGI-I), Cognitive interviews, Content validity

## Abstract

**Objective:**

To establish content validity of a single-item, migraine-specific symptom severity questionnaire for completion by migraine patients, key family members (KFMs) of migraine patients, and Healthcare Professionals (HCPs) who treat migraine patients.

**Background:**

Migraine is a common disabling primary headache disorder with high prevalence and significant socioeconomic burden and personal impacts. There is a need for a global assessment of migraine symptom severity to evaluate potential new therapies from multiple perspectives.

**Methods:**

The migraine Global Impression Item (mGI-I) was drafted and tested in a non-interventional, qualitative study comprising telephone interviews with 15 migraine patients, 15 KFMs of migraine patients, and 15 migraine treating HCPs. The mGI-I was drafted with two different item stem options and two different response scale options to ask about the patient’s migraine from the perspective of each respondent. Cognitive interviews were conducted to test comprehensiveness, clarity and ease of completion of the different versions of the mGI-I iteratively in three sequential waves of respondents.

**Results:**

Revisions were made to the draft mGI-I after Wave 1 and Wave 2 of the interviews. Changes were made to simplify the item stem (removing unnecessary text), make language more patient-friendly (e.g. use of “migraine attack”), and add clarity to the item stem for consistent interpretation (include descriptive language of migraine attacks). Across both waves there was a preference for a 5-point response scale compared to a 7-point scale. In Wave 3, all respondents found the revised instructions, item stem, and 5-point response scale comprehensive, easy to understand and to answer. No further changes to the mGI-I were made after Wave 3.

**Conclusions:**

This qualitative study of 45 total respondents across 3 subpopulations, established the content validity and appropriateness of the mGI-I in migraine patients, KFMs, and migraine-treating HCPs. The study specifically confirmed that the mGI-I is comprehensive, easily understood and answered for each respondent population.

**Supplementary Information:**

The online version contains supplementary material available at 10.1186/s12883-022-02626-0.

## Background

Migraine is a neurological disorder characterized by recurrent headache episodes that are often associated with nausea, vomiting, photophobia and phonophobia. Population studies indicate that migraine prevalence is between 2.6 and 21.7%, with an average of around 12% [1]. In the United States, migraine and severe headache have a prevalence of 15.3% in the adult population over a three-month period, a figure that has reportedly been stable over a 19-year period [2]. Migraine may be either episodic or chronic, both of which have significant socioeconomic burden and personal impacts (ICHD-3, 2018) [3]. Migraine ranks in the top 10 causes of years lived with disability worldwide [4] and is the leading cause of this parameter in individuals under 50 years old [5].

Patients with chronic migraine experience substantially greater impact on daily activities, higher direct medical costs, greater overall health care resource utilization, reduced health-related quality of life (HRQOL), and higher rates of comorbidities compared with those with episodic migraine [6, 7].

In a recent retrospective, cross-sectional study in France, Germany, Italy, Spain, and the United Kingdom, it was found that migraine sufferers (≥ 4 headache days per month) experienced poorer HRQOL, greater work productivity loss, higher activity impairment, and higher health care resource utilization than nonmigraine controls [8]. Another survey, of 11,266 migraine patients from 31 countries, found that 74% spent time in darkness/isolation due to migraine (for an average of 19 h /month), and 85% reported negative aspects of migraine [9].

### The appropriateness of a global assessment

Multiple scales have been developed to assess patient- or physician-reported migraine severity, including the Migraine Disability Assessment Scale (MIDAS) [10], the Headache Impact test (HIT-6) [11], the Henry Ford Hospital Disability Inventory (HDI) [12], and the Migraine Severity (MIGSEV) Scale [13]. These measures are lengthy, focus on migraine-related disability, and are sensitive to recall bias based on the specificity of the concept of interest. The Global Assessment of Migraine Severity (GAMS) was developed as a single-item global scale to assess patients’ perception of their disease severity [7]. Other than MIGSEV, all of these scales are based on the patient’s perspective of migraine and related symptoms. Furthermore, the perception of family members is not incorporated in any of these assessments, and none allows direct comparisons between patient and physician perceptions of migraine. A simple global scale utilizing similar wording and the same response options for completion by patients, family members, and physicians would further our understanding of treatment impact on migraine frequency and severity from perspectives in addition to that of the patient.

Given the need for comprehensive, yet simple scales that provide data across these three subpopulations – patients, key family members (KFMs) and migraine treating healthcare providers (HCPs) – there could be broad utility for a global scale.

### Development of the global assessment

The Clinical Global Impression of Improvement (CGI-I) item of the Clinical Global Impression set of brief assessment tools is a widely used global measure of change in severity of people with multiple chronic conditions, including migraine and other chronic disorders [14]. This is a single item with responses recorded on a 7-point response scale (Table 1). However, this has been criticized for being too generic [15], and only generates information from the HCP perspective. It does offer a well-established basis from which to develop new scales which are migraine specific, and which ask patients, KFMs, and HCPs to independently report on migraine severity.

**Table 1:** The original CGI-I [14]

Original CGI-I		
Assessor	Item Stem	Response Scale
To be completed by the clinician:	Rate total improvement whether or not, in your judgement, it is due entirely to drug treatment. Compare to his condition at admission to the project, how much has he changed?	0 = Not assessed 1 = Very much improved 2 = Much improved 3 = Minimally improved 4 = No change 5 = Minimally worse 6 = Much worse 7 = Very much worse

*CGI-I* Clinical global impression of improvement

The initial draft version of the mGI-I (Table 2) was adapted from the CGI-I. It was developed based on domain knowledge of migraines and clinical outcomes assessment development practices. Prior to including any new instrument in a clinical research study, it is important to establish its content validity within its targeted context of use [16, 17].

**Table 2:** mGI-I draft version 1.0

	Modified CGI-I (initial Wave 1 version)^a^		
	Patient	KFM	HCP
**Instruction**	The next question asks about your migraines at the moment. Please choose one answer. There is no right or wrong answer – we are interested in how you feel.	The next question asks about your family member’s migraines at the moment. Please choose one answer. There is no right or wrong answer – we are interested in how you feel.	The next question asks about your patient’s migraines at the moment. Please choose one answer. There is no right or wrong answer – we are interested in how you feel.
**mGI-I**	Compared to the migraines you were experiencing before starting your latest treatment, how are your migraines now? ° Very much improved ° Much improved ° A little improved ° No change ° A little worse ° Much worse ° Very much worse	Compared to the migraines your family member was experiencing before starting their latest treatment, how are their migraines now? ° Very much improved ° Much improved ° A little improved ° No change ° A little worse ° Much worse ° Very much worse	Compared to the migraines your patient was experiencing before starting their latest treatment, how are their migraines now? ° Very much improved ° Much improved ° A little improved ° No change ° A little worse ° Much worse ° Very much worse
**Alternate version mGI-I stem**	• Compared to the migraines you were experiencing before starting your latest treatment, how are your migraines now? (please consider the number of migraine days per month, the physical impairment associated with migraine, and the impact of migraine on everyday activities)	• Compared to the migraines your family member was experiencing before starting their latest treatment, how are their migraines now? (please consider the number of migraine days per month, the physical impairment associated with migraine, and the impact of migraine on everyday activities)	• Compared to the migraines your patient was experiencing before starting their latest treatment, how are their migraines now? (please consider the number of migraine days per month, the physical impairment associated with migraine, and the impact of migraine on everyday activities)
**Alternate version mGI-I response options**	° Much improved ° A little improved ° No change ° A little worse ° Much worse	° Much improved ° A little improved ° No change ° A little worse ° Much worse	° Much improved ° A little improved ° No change ° A little worse ° Much worse

*mGI-I* Migraine global impression item, *KFM* Key family member, *HCP* Health care provider

^a^This initial modified version was tested in Wave 1 and then refined based on iterative feedback

## Methods

The objective of this study was to iterate and establish content validity of a single-item, migraine-specific questionnaire, modified from the CGI-I item of the Clinical Global Impression set of brief assessment tools [14, 18]. This questionnaire – the mGI-I – was designed to assess perspectives of improvement or worsening in migraine symptoms following a therapeutic intervention.

Patients were aged 18 years or above, with a history of migraines lasting 1 year or more, currently on preventative treatment for their migraines, and experiencing four or more 4 migraines/month in the last 3 months.

The KFM population was identified by the enrolled patient sample via the patient screening form, which asked the patient to nominate “the person in your life that you interact with the most on a day-to-day basis. This person should have a deep understanding of the impact migraine has on you. They should also be able to provide their perspective of change in your migraines over time. The person you choose to nominate can be anyone 18 years or older such as your spouse/partner, your daughter or son, a parent, sibling, other relative or the person you are in a defacto relationship with.”

Migraine Treating HCPs were licensed and practicing providers (i.e., neurologist, headache specialist, primary care physician, nurse practitioner, physician assistant) in the US, with a minimum of 5 years of clinical experience, and currently treating at least 30 migraine patients per month.

### Study design

Three different versions of the mGI-I were designed for completion by 3 subpopulations; migraine patients, KFMs of migraine patients, and HCPs who treat migraines.

This non-interventional, qualitative study involved one-on-one telephone interviews conducted with a total of 15 migraine patients, 15 KFMs of migraine patients, and 15 migraine treating HCPs (neurologists, headache specialists, and primary care physicians). Per established literature and the simplicity of the instrument, a sample of 15 interviews in each population was planned and resulted in sufficient data to establish confidence in the comprehension, relevance and applicability of the instrument [19, 16]. The interviews were conducted in three sequential waves (groups), from August 2020 through November 2020. This approach allowed the scale to be iterated in between the waves.

Wave 1 involved 13 interviewees (five HCPs, four patients, four KFMs); Wave 2 had 15 interviewees (five HCPs, five patients, five KFMs); and Wave 3 had 17 interviewees (five HCPs, six patients, six KFMs). The mGI-I was tested with two options for the item stem (one with and one without descriptive text within parentheses) and two different response scales (one with 5 response options and one with 7 response options) (Table 2). Inclusion/exclusion criteria for each respondent subpopulation (used in all three waves) are summarized in Table 3.

**Table 3:** Inclusion/exclusion criteria

Population	Inclusion criteria	Exclusion criteria
Patient	• Adults aged ≥18 years old • History of migraines ≥12 months according to International Classification of Headache Disorders III (ICHDIII) • Patients currently on preventative treatment for their migraines • ≥4 migraines/month in the last 3 months • Must identify a KFM that meets criteria/definition (and that KFM must consent) • Able and willing to provide informed consent and participate in one-on-one telephone interviews in English • Able and willing to obtain clinical confirmation of diagnosis from their treating physician	• History of cluster headache or hemiplegic migraine headache • Active chronic pain syndromes (such as fibromyalgia and chronic pelvic pain) • Taken an opioid or butalbital-containing analgesics on ≥4 days per month for any indication in any month during the two months prior to the start of the study • History of major psychiatric disorder or drug use that would impact the subject’s ability to complete the consent or interview procedures
KFMs	• Target is to be the KFM of an enrolled patient (but this is not a requirement) ° The KFM population was identified through the enrolled patient sample via the patient screening form with the following language: ▪ Part of this is asking you to nominate your “key family member” - the person in your life that you interact with the most on a day-to-day basis. This person should have a deep understanding of the impact migraine has on you. They should also be able to provide their perspective of change in your migraines over time. The person you choose to nominate can be anyone 18 years or older such as your spouse/partner, your daughter or son, a parent, sibling, other relative or the person you are in a defacto relationship with. • Age ≥ 18 years old • Able and willing to provide informed consent and participate in one-to-one interviews	There were no specified exclusion criteria for the KFMs.
Migraine Treating HCPs	• Licensed and practicing HCPs (i.e., neurologist, headache specialist, primary care physician, nurse practitioner, physician assistant) in the US • Minimum of 5 years of clinical experience • Currently treating at least 30 migraine patients/month • Able and willing to provide informed consent and participate in one-on-one telephone interviews in English	There were no specified exclusion criteria for the HCPs.

IQVIA moderators, trained and experienced in qualitative interviewing, conducted the 30-60 min cognitive interviews with the respondents, including completing the mGI-I and providing feedback on the instructions and items with regard to relevance, comprehensiveness and clarity. IQVIA is a world leader in using data, technology, advanced analytics, and expertise to help customers drive healthcare forward.

The findings from each wave of interviews were reviewed to assess whether there were any substantial issues that would warrant changes to the instructions and/or mGI-I item. Interview respondents were compensated with a fair-market honorarium after completion of the interview.

The interview guide and all processes and respondent-facing documents were approved by the New England IRB (#20200306) before research was initiated.

#### Recruitment procedures

Respondents were recruited by third-party vendors. Patients and KFMs were recruited by Global Perspectives (Norwich, UK) from the company’s study database, patient associations, and social media. Patients and KFMs provided paper informed consent or completed an electronic consent form. HCPs were recruited by Atheneum (Berlin, Germany) using the company’s panel of HCPs who agree to participate in research studies. All respondents (patients, KFMs, HCPs) were from the USA.

#### Interview process

Interviews were conducted via telephone using the Webex screen share platform, using a semi-structured interview guide that facilitated the cognitive interviews. The questions in the CI portion were developed using best practice guidelines [16] and presented in an electronic format. Respondents were asked to identify any areas of the mGI-I scale that were confusing, problematic or not relevant. Table 4 illustrates examples of some of the topics explored to address the study objectives. The interviews were audio recorded with the respondent’s consent.

**Table 4:** Example topics in the interview guide

Interview Topic	Objective
• Asked if there was anything unclear about the instructions or the item • Asked if the question was relevant to their experience with migraine • Asked if they could put the question into their own words • Asked to answer the item and if they were able to easily find a response option that fit their experience with migraine • Asked what they considered when answering the item	To document evidence of content validity for the mGI-I for each of three migraine-related subpopulations: migraine patients, KFMs, and treating HCPs
• Asked how they interpret the difference between response options (e.g., Much Improved vs A Little Improved as well as Much Worse vs. A Little Worse) • Asked what changes they needed to have occur to have experienced improvement or worsening	To document how respondents defined improvement/worsening in migraine severity
• Asked their preference (and rationale for preference) between the item stems • Asked their preference (and rationale for preference) between the response scales	To aid in selection of most appropriate and comprehensive item to the target population

#### Data management and analysis

Audio recordings from the interviews were transcribed by a professional transcription service; any personal identifying information was removed from the transcripts. Management and analysis of interview data was completed following each wave of the cognitive interviews, with the rationale for any change recorded and any revisions for the next wave documented. All data were summarized using descriptive statistics.

## Results

### Respondent characteristics

The total sample comprised 45 respondents; 15 patients, 15 KFMs and 15 HCPs. All KFMs were family members of enrolled patients. Table 5 shows the characteristics of respondents included in the study. The mean age of the enrolled patients was 37.7 years, and 80% (*n* = 12) of patients were female. The KFMs (73% male) included husbands, brothers, daughters, and mothers. The HCPs included 9 neurologists and headache specialists and 6 primary care physicians.

**Table 5:** Patient demographic and clinical characteristics

**Patient demographic characteristics (*n*** **= 15)**	
Age (Years):	
Mean (SD)	37.7 (9.4)
Median	39
Range	25-57
Gender:	
Male	3 (20%)
Female	12 (80%)
Education:	
Some College	2 (13%)
Bachelors	10 (67%)
Masters	2 (13%)
Doctorate	1 (7%)
**Patient clinical characteristics (*n*** **= 15)**	
Medications	
Previously or currently taking CGRP inhibitor	9 (60%)
Self-reported migraine type	
Chronic	7 (47%)
Episodic	8 (53%)
**KFM demographic characteristics (*n*** **= 15)**	
Gender:	
Male	11 (73%)
Female	4 (27%)
Relationship	
Daughter	2 (13%)
Mother	2 (13%)
Husband	9 (60%)
Brother	2 (13%)
**HCP clinical specialties and professional experience (*n*** **= 15)**	
Specialty	
Neurologist / Headache Specialist	9
Primary Care Physician	6
Migraine patients seen per month	
Mean (SD)	101.5 (116.4)
Median	45
Range	15-400
Experience in practice (Years)	
Mean (SD)	17.3 (8.0)
Median	15
Range	5-30
Geographic location of practice in the USA	
Northeast	8 (53%)
Southeast	1 (7%)
Midwest	5 (33%)
West	1 (7%)

### Wave 1 cognitive interview results

The mGI-I scales shown in Table 2 were tested in Wave 1 with four patients, four KFMs, and five HCPs. The instruction screen was tested along with two versions of the item stems and two versions of the response options. The first wave was used to understand the comprehension of the mGI-I and respondent preference between the two versions of the item stem and response options.

Detailed insights from Wave 1 interviews included the following:

- Three out of four patients (but no KFMs or HCPs) suggested changing the term “migraine” to “migraine attack” throughout the mGI-I. This term was added as a probe for Wave 2.
- All five HCPs found the instructions to be clear, but said the phrase, “we are interested in how you feel” was unnecessary. This change was implemented for Wave 2. There were no issues with the instructions for the patients or KFMs.
- Three out of four patients, four out of four KFMs, and four out of five HCPs preferred the alternative item stem with additional descriptive language in parentheses. The alternate version with the descriptive parentheses only was tested in Wave 2. Respondent feedback included “*The extra language was helpful, because migraines are multi-faceted.” –* KFM
- Two patients suggested changing “physical impairment” to “pain severity.” In the alternative item stem, a probe was added to the discussion guide to explore this point in Wave 2.
- Three out of four patients, two out of four KFMs, and three out of five HCPs preferred the 5-point response scale. Both response scales were taken forward to Wave 2.

### Wave 2 cognitive interview results

Five patients, five KFMs, and five HCPs comprised Wave 2. Detailed insights from Wave 2 interviews for the revised mGI-I scales are provided below:

- Three out of five patients, three out of five KFMs, and three out of five HCPs use and preferred the term “migraine attack” to “migraine.”
- The six respondents preferring the term “migraines” stated they could still answer the item accurately if “migraine attack” was used. “Migraine attack” was carried forward to Wave 3; all 3 mGI-I scales were changed to refer to “migraine attacks” instead of “migraines.”
- All patients, KFMs, and HCPs in Wave 2 found the instructions to be clear.
- The alternative item stem was changed to refer to “pain severity” instead of “physical impairment”; five out of five patients, two out of five KFMs, and four out of five HCPs preferred the term “pain severity” to “physical impairment” in the alternative item stem.
- Two out of five patients, three out of five KFMs, and four out of five HCPs preferred the 5-point response scale option, which was retained for testing in Wave 3. Respondent feedback included *“I prefer the 5-point scale. With the 7-point scale, the additional response options are not common and don’t play out clinically.”* – HCP

### Wave 3 cognitive interview results

The revised mGI-I scales were tested in Wave 3 with six patients, six KFMs, and five HCPs. Only the item stem with additional descriptive language in parentheses and the 5-point response scale were tested. No issues were identified with the instructions, item, or 5-point response scale. All respondents found them easy to understand and to answer. No further changes were proposed or made.

A summary of the Item Tracking Matrix (Table 6) documents the significant feedback from the respondents and rationale/decisions for changes to the mGI-I scales from the waves of interviews.

**Table 6:** Summary of modifications from Item Tracking Matrix

	Instructions	Item Stem & Alternative Item Stem	Response Options
**Wave 1**			
Patient	Add probe to change from “migraine” to “migraine attack”	Only test alternative item stem” in next wave. Add probe to discussion guide to explore potential change of “physical impairment” to “pain severity”	No change
KFM	Add probe to change from “migraine” to “migraine attack”	Only test alternative item stem in next wave. Add probe to discussion guide to explore potential change of “physical impairment” to “pain severity”	No change
HCP	Add probe to change from “migraine” to “migraine attack” Removed the phrase “we are interested in how you feel”	Only test alternative item stem in next wave. Add probe to discussion guide to explore potential change of “physical impairment” to “pain severity”	No change
**Wave 2**			
Patient	Change the term “migraine” to “migraine attack” to better connect with patients	Change the term “migraine” to “migraine attack” to better connect with patients. Change the term “physical impairment” to “pain severity”. Continue to only test the alternate item stem with descriptive	Only continue to test the majority preferred 5-point scale (based on the Wave 2 feedback from all respondents as well as having 17 respondents preferring the 5-point scale versus only 11 respondents preferring the 7-point scale)
KFM	Change the term “migraines” to “migraine attacks”	Change the term “migraines” to “migraine attacks” Change the term “physical impairment” to “pain severity”. Continue to only test the alternate item stem with descriptive.	Only continue to test the majority preferred 5-point scale (based on the Wave 2 feedback from all respondents as well as having 17 respondents preferring the 5-point scale versus only 11 respondents preferring the 7-point scale)
HCP	Change the term “migraines” to “migraine attacks”	Change the term “migraines” to “migraine attacks” Change the term “physical impairment” to “pain severity”. Continue to only test the alternate item stem with descriptive.	Only continue to test the majority preferred 5-point scale (based on the wave 2 feedback from all respondents as well as having 17 respondents preferring the 5-point scale versus only 11 respondents preferring the 7-point scale)
**Wave 3**			
Patient	No further changes	No further changes	No further changes
KFM	No further changes	No further changes	No further changes
HCP	No further changes	No further changes	No further changes

### Final versions of the mGI-I

Following the three waves of cognitive interviews, the mGI-I scales were finalized, having been demonstrated to be understandable, relevant and easy to complete by the final wave of study respondents. In addition, all subpopulations were able to distinguish between all response options and select answers with ease. Although several symptoms are associated with migraine attacks, each subpopulation was able to provide the rationale for selecting a response with the 5-point scale. Overall, patients selected a response primarily based on a decrease or increase in frequency and pain severity, but symptoms such as nausea, brain fog and light sensitivity were also considered relevant factors even though not always consistent with each headache. KFMs considered impact on daily activities when selecting a response to indicate improvement or worsening. HCPs were aligned with the main drivers identified by patients (i.e., frequency and pain severity) and KFMs (i.e., daily activities); however, HCPs indicated that they would often defer to their patients when determining improvement or worsening of migraine attack severity. Also, HCPs would ask patients about any improvement or worsening related to sensitivity to light, noise, touch, an increase or decrease in the use of therapies, and any increase or decrease in the duration of migraines.

Based on all the qualitative data, the final scales (included in supplemental documents) are considered content valid and ready for implementation.

## Discussion

The results from the three waves of cognitive interviews suggest that the mGI-I is clear and relevant to patients with migraine, their KFMs and treating HCPs. All respondents confirmed that the mGI-I captures the most important concepts related to migraine attacks. It is worth discussing the three most significant decisions in the development and iteration of the scale.

First, the term migraine attacks was preferred over “migraines” to describe the patient experience of their migraine. This is consistent with a September 2020 American Academy of Neurology (AAN) updated quality measurement set for headache, which uses the term “migraine attack” rather than “migraine” when measuring outcomes [20].

Second, the question, while intended to be a global measure of change in disease severity, includes instructions within the item stem to consider the number of migraine attacks, degree of pain and the impact on everyday activities. These could be considered as individual items as proposed by the U.S. Food and Drug Administration [21], but the qualitative data supported the idea that all contributed to an overall assessment of severity. However, all items were not considered as equally important by all respondents, making the creation of a scoring algorithm using multiple items difficult. A single item is therefore considered appropriate. All patients, KFMs, and HCPs confirmed that they considered the frequency, pain severity, and impact on daily activities due to migraine attacks when completing the final version of the scale and were able to select an appropriate response to describe improvement or worsening.

Third, the 5-point response scale was preferred by the majority of respondents when they were offered the choice between 5- and 7-point response scales.

Based on this research, a scale that is clinically appropriate and which has demonstrated content validity across three subpopulations has been validated. Administering the mGI-I meets the need for a simple scale in various settings and allow for the exploration of similarities and differences among the identified subpopulations.

### Limitations

The limitations of this research include the sample being US only, the recruitment being through vendors so that it was a convenience sample, and the relatively small sample size that is typical of qualitative research. The analysis was descriptive based on thematic coding. While these are limitations overall, this project followed best practices for conducting qualitative research. Quantitative evaluation of the scales has not yet been conducted.

## Conclusions

This qualitative study of 45 respondents has established the content validity and appropriateness of the mGI-I in patients with chronic or episodic migraines, patients’ KFMs, and migraine-treating HCPs. The study specifically confirmed that the modified version of the CGI-I scale (now the mGI-I) is comprehensive and easily understood and answered by these subpopulations. This evidence confirms that these subpopulations of interest can understand and find the mGI-I relevant to the experience of migraine. Regardless of how the respondent came to a response selection, all respondents easily answered the questions about the frequency, pain severity and daily impact of activities specific to their migraine condition.

## Supplementary information

**Additional file 1: Figure S1.** FinalmGI-I scale for patients (This is review copy, do not use without permission). **Figure S2.** Final mGI-I scale for KFMs (This is review copy, do not use without permission). **Figure S3.** Final mGI-I scale for HCPs (This is review copy, do not use without permission).

## Data Availability

The datasets used and/or analyzed during the current study are available from the corresponding author on reasonable request.

## Abbreviations

KFMs: Key family members of migraine patients
HCPs: Healthcare Professionals
mGI-I: Migraine Global Impression Item
HRQOL: Health-related quality of life
MIDAS: Migraine Disability Assessment Scale
HIT-6: Headache Impact test
HDI: Henry Ford Hospital Disability Inventory
MIGSEV: Migraine Severity Scale
CGI-I: Clinical Global Impression of Improvement
