# Supplementary material for: Establishing content validity for the migraine Global Impression Item (mGI-I) assessment: a modified single-item migraine symptom severity questionnaire
Source: BMC Neurol. 2022 Mar 18;22:103. doi: 10.1186/s12883-022-02626-0 (PMC8932152; doi:10.1186/s12883-022-02626-0)

This supplement document is NOT subject to a Creative Commons License.

Figures S1-S3 Final mGI-scales © [2020] Amgen Inc. All rights reserved.

**FIGURE S1: Final mGI-I scale for patients** (This is review copy, do not use without permission)


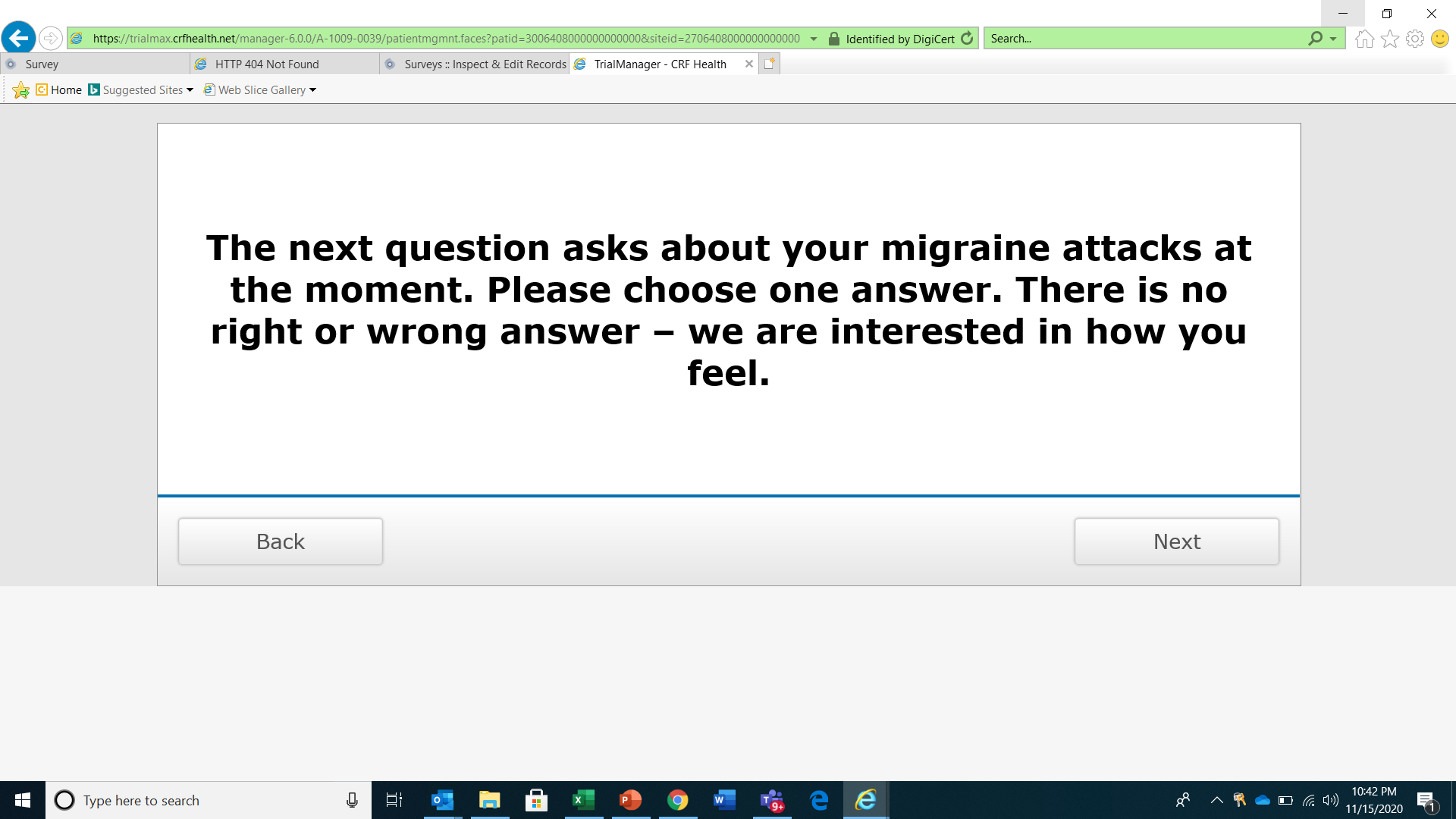


**Review copy do not use without permission**


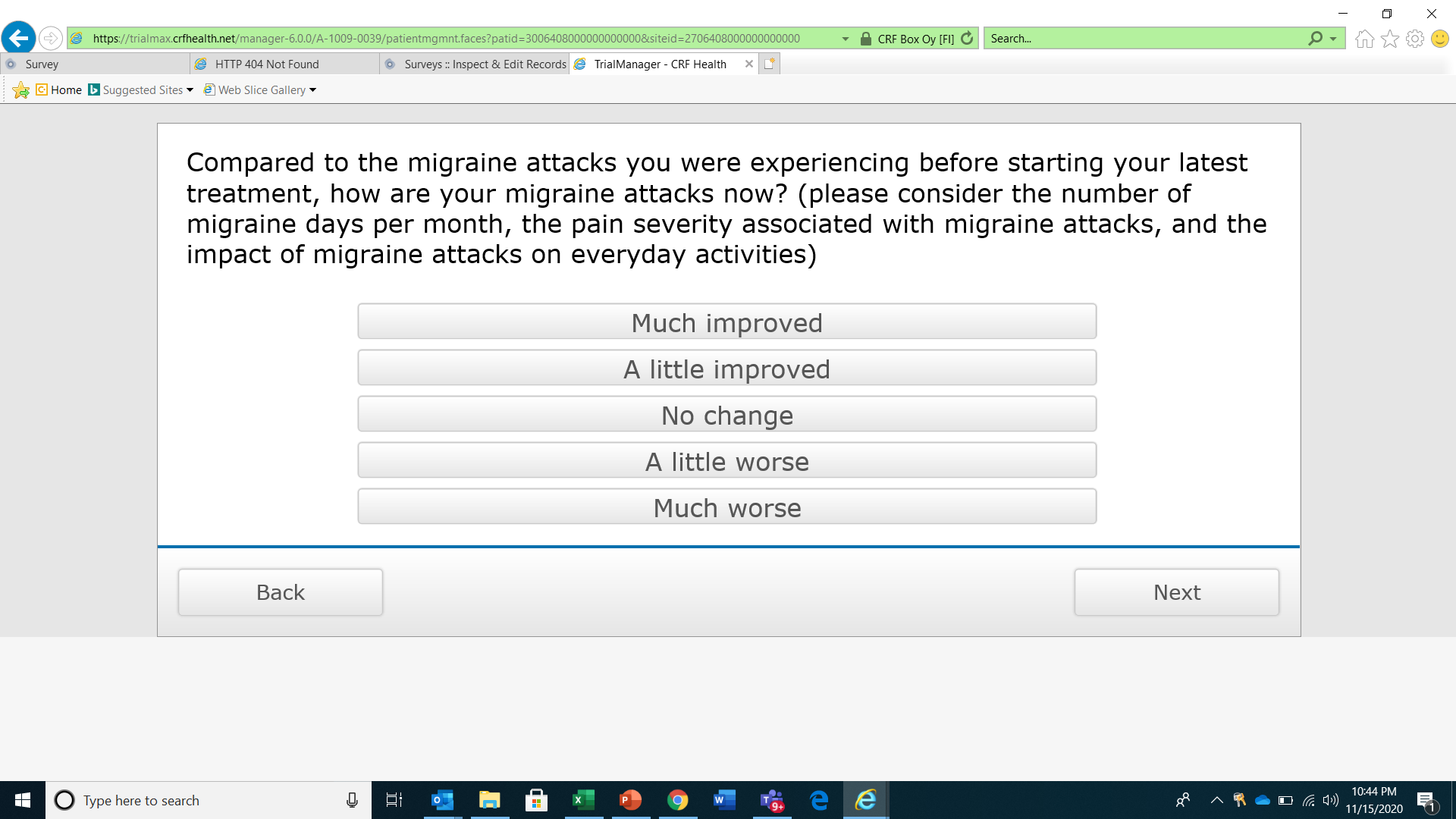


**FIGURE S2 : Final mGI-I scale for KFMs** (This is review copy, do not use without permission)


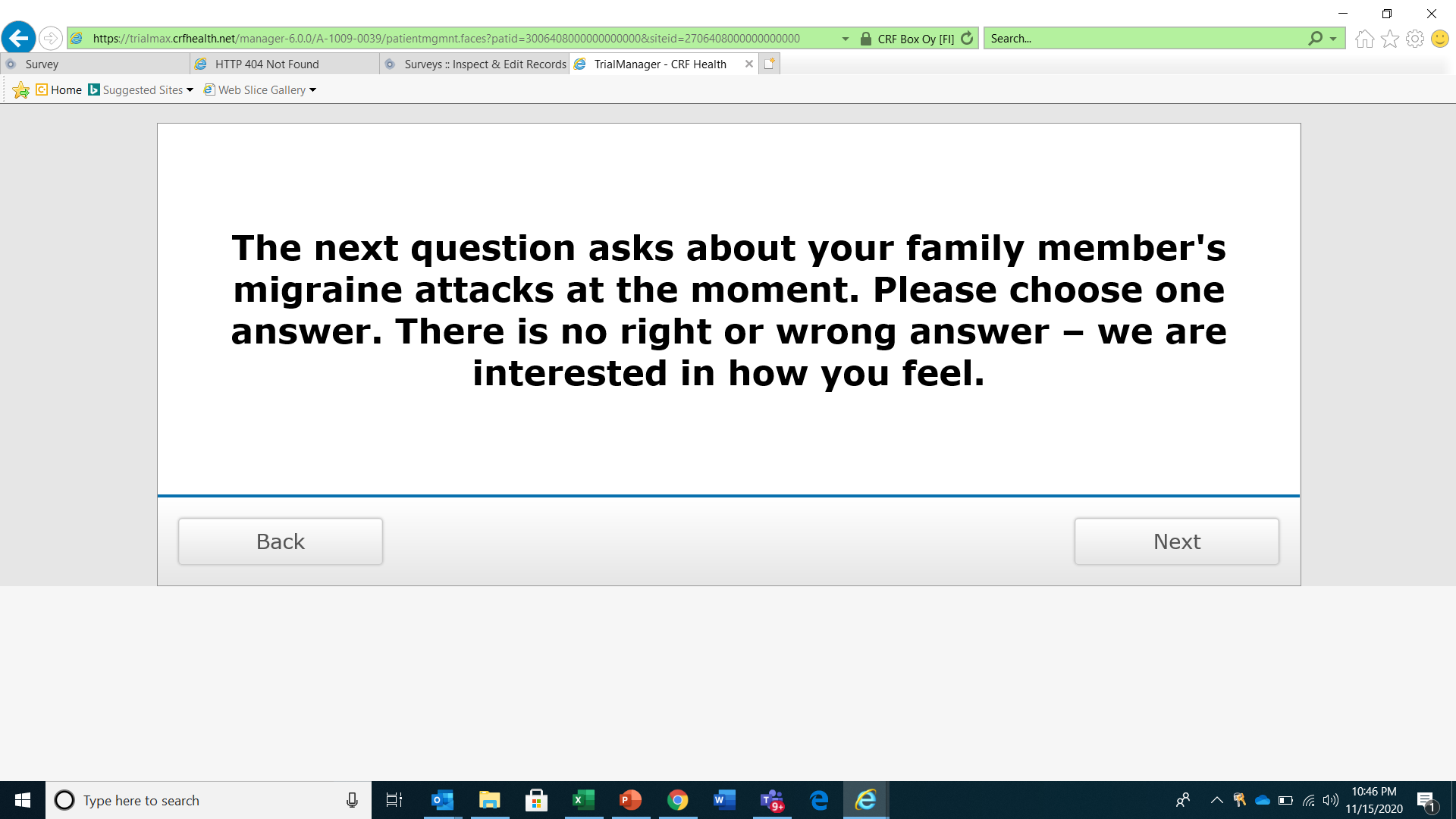


**Review copy do not use without permission**


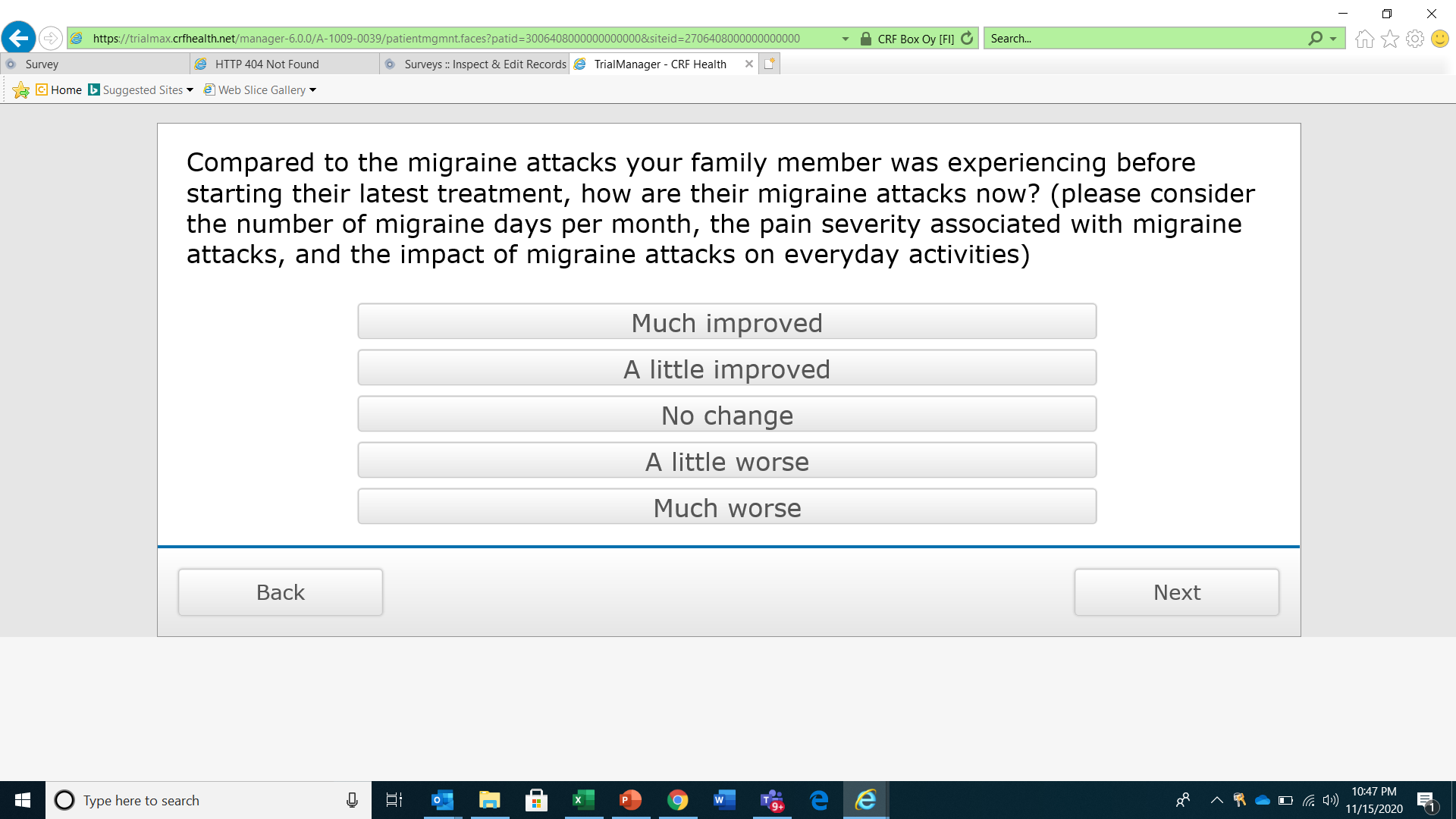


**FIGURE S3: Final mGI-I scale for HCPs** (This is review copy, do not use without permission)


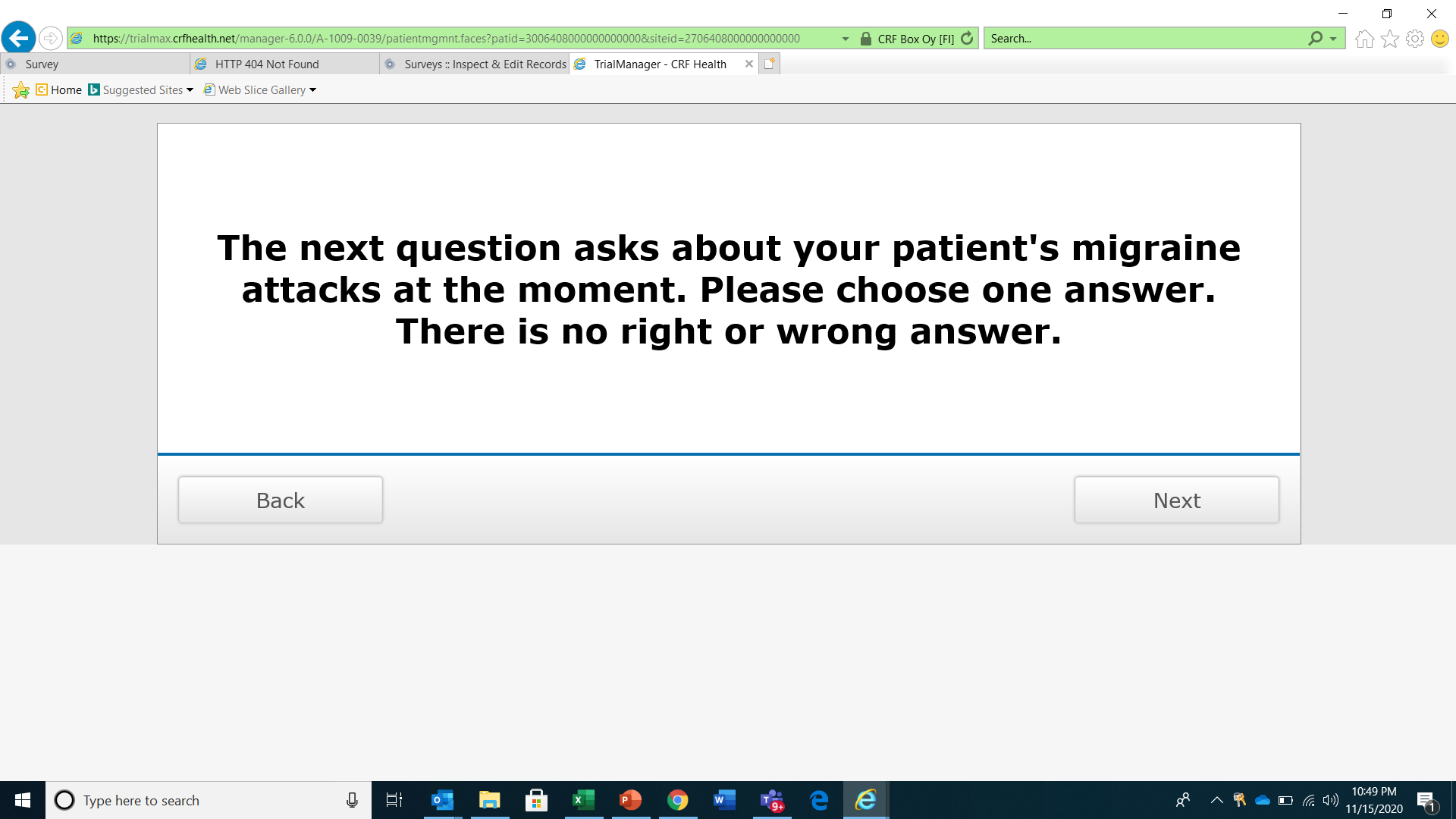


**Review copy do not use without permission**


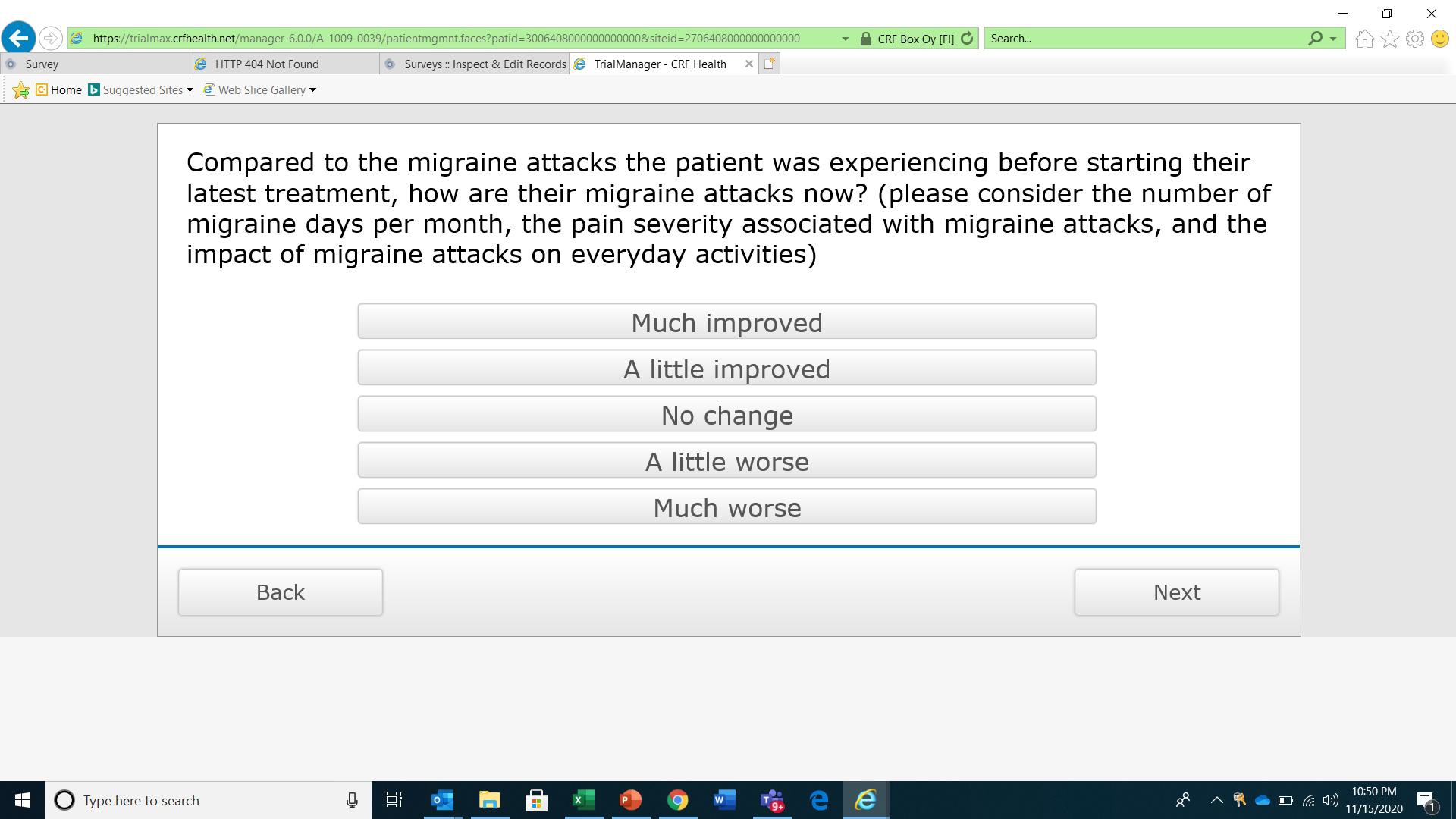

Supplement: Supplementary file 1 — Additional file 1: Figure S1. FinalmGI-I scale for patients (This is review copy, do not use without permission). Figure S2. Final mGI-I scale for KFMs (This is review copy, do not use without permission). Figure S3. Final mGI-I scale for HCPs (This is review copy, do not use without permission). [file 12883_2022_2626_MOESM1_ESM.docx]
